# High Prevalence of a Novel Circovirus in the European Hedgehog (*Erinaceus europaeus*), a Common Species in Decline

**DOI:** 10.1155/2024/4670252

**Published:** 2024-11-27

**Authors:** Kevin P. Mulder, Frank Pasmans, Filip van Nieuwerburgh, Naomi Terriere, Moira Kelly, Seline Bregman, Elin Verbrugghe, An Martel

**Affiliations:** ^1^Wildlife Health Ghent, Faculty of Veterinary Medicine, Ghent University, Merelbeke, Belgium; ^2^Laboratory of Pharmaceutical Biotechnology, Faculty of Pharmaceutical Sciences, Ghent University, Ghent, Belgium

**Keywords:** cfDNA, hedgehog diphtheric disease, metagenomics, pathogen discovery, phylogenetics, wildlife disease

## Abstract

Hedgehog (*Erinaceus europaeus*) declines in western Europe have been associated with the emergence of Hedgehog diphtheric disease (HDD), with a probable multifactorial, yet unidentified etiology. We used metagenomic sequencing of cell-free DNA (cfDNA) in hedgehog blood to identify possible causes of HDD. We detected a novel circovirus species in the European hedgehog, providing the first record of a circovirus within the mammalian order Eulipotyphla. The novel circovirus genome exhibits the characteristic circovirus structure, including a functional replicase (REP) and capsid (CAP) gene. Phylogenetic analysis placed all four detected genomes in a monophyletic clade, most closely related to sequences isolated from dogs. Subsequent PCR-based screening of 188 hedgehog liver samples demonstrated a high prevalence (61%) of this circovirus in hedgehogs brought to wildlife rescue centers, however, without any significant association with HDD. Since circoviruses are well known to interfere with host immunity across mammalian and avian taxa, the high level of circovirus detection in hedgehogs warrants further research into the role of this novel virus in hedgehog health.

## 1. Introduction

Our understanding of biodiversity is often constrained by the observational methods employed, and this limitation is particularly evident in the microbial world. Metagenomic barcoding of environmental samples has proven crucial for discovering novel species and strains, as well as quantifying the abundance of fungal and bacterial pathogens [1, 2]. However, viruses remain a frequently overlooked component of the natural world, as their highly variable genomes pose challenges for conventional barcoding techniques that rely on primer development [3]. Metagenomic shotgun sequencing offers a solution, directly sequencing all available DNA within a sample without reliance on primers [2]. However, mixed host-pathogen samples necessitate increased sequencing depth, as larger host genomes can dominate the data. Cell-free DNA (cfDNA), with its reduced host DNA content and enrichment of both bacterial and viral DNA, provides a promising avenue for novel pathogen discovery within host systems [4–6].

The European hedgehog (*Erinaceus europaeus*) is an iconic species found across much of western and northern Europe. It survives well in anthropogenic habitats and thus contact with humans is comparatively common [7], and they are often found by members of the public and brought to local animal rescue centers [8]. Extensive research exists on their genetics [9], ecology [10], and pathogens [11–15]. The large number of identified pathogens and its close contact with humans highlights hedgehogs as potential sources of zoonotic diseases [14, 16, 17).

A recent outbreak of Hedgehog diphtheric disease (HDD) causing ulcerative dermatitis in hedgehogs across Belgium is associated with the common bacterium *Corynebacterium ulcerans* [13, 18]. As it is not a newly introduced pathogen and there are no signs of a new virulent lineage in these populations [13], the disease outbreak is likely multifactorial and other pathogens and environmental factors may also be impacting hedgehog health and increasing susceptibility to this toxigenic bacterium [18]. To investigate potential novel or undetected pathogens, we employed metagenomic sequencing of cfDNA extracted from the blood of hedgehogs with HDD. Our objectives were to identify novel infectious agents by means of metagenomic analyses, reconstruct the evolutionary context of any newly found pathogens, and explore their potential correlations with HDD.

## 2. Methods

### 2.1. Sampling and Library Preparation

Six hedgehogs with severe HDD were brought to a wildlife rescue center (Geraardsbergen, Flanders, Belgium) by members of the public from the surrounding area in June 2021 (Supporting Information 1: Table S1). Due to their severe condition, euthanasia was deemed necessary. Exsanguination via cardiac puncture was performed after isoflurane anesthesia, followed by EUTHASOL—pentobarbital sodium and phenytoin sodium solution. Collection tubes were immediately inverted five times to ensure blood-anticoagulant mixing. Samples were centrifuged (4750 rpm, 30 min) and plasma was transferred to polypropylene tubes and stored at −70°C. The collection area was thoroughly cleaned and disinfected before and after each animal to ensure a sterile environment. Handlers wore protective gloves and gowns to safeguard themselves and prevent crosscontamination. Sterile equipment was used and aseptic techniques were applied to prevent pathogen introduction.

Upon thawing, samples were recentrifuged to minimize cellular contamination. DNA was extracted from 1 ml of plasma using the MagMAX cfDNA isolation kit (ThermoFisher Scientific) according to the manufacturer's protocol and eluted in 11 µl of buffer. DNA concentration and fragment size distribution were assessed using a high sensitivity DNA chip on a 2100 Bioanalyzer (Agilent Technologies). Illumina libraries were prepared using the NEBNext Ultra II library prep kit (NEB) with unique dual indices and six cycles of polymerase chain reaction (PCR) amplification. PCR products were purified with Ampure XP beads (Beckman Coulter), and library profiles were assessed on a Bioanalyzer with a high sensitivity DNA chip. Library concentrations were determined via qPCR. Samples were pooled equimolarly, and adapter dimers were removed from the final pool using an E-gel EX 2% agarose gel (Life Technologies) and the Zymo DNA Gel recovery kit (Zymo Research). Libraries were sequenced on a HiSeq3000 lane using paired-end 150-bp reads.

### 2.2. Metagenomic Analyses

To classify the metagenomic reads we used the taxonomic read classifier Kraken (v2.1.3) which uses a k-mer based reference index to classify each read based on publicly available genomic data [19]. It applies the NCBI taxonomic database to order all classified reads based on the taxonomic divisions. Sequencing data were first demultiplexed by barcode by the sequencing facility, and all subsequent computations were conducted on the UGent High Performance Cluster (HPC). Sequences were filtered and trimmed using Trim_Galore v0.6.4 ([20, 21]; -paired-quality 20 -max_n 1 -length 140). To remove host reads we built a custom Kraken2 database using the *E. europaeus* reference genome (mEriEur2; NCBI RefSeq assembly GCF_950295315.1) and ran the full dataset through this database using default parameters. Only reads that were not classified as belonging to the host were kept as input for novel viral and bacterial pathogen discovery.

Non-host reads were classified using the Kraken2 database which includes all published viral, bacterial, fungal, and protozoan genomes (PlusPF index version 20240112) applying default parameters but increasing the classification threshold to reduce spurious hits (--confidence 0.03 --minimum-hit-groups 5; [22]). Unclassified reads were removed and we produced kronaplots using KronaTools 2.8.1 [23] to visualize bacterial and viral composition.

### 2.3. Circovirus Phylogenetic Reconstruction

**Figure 1:**
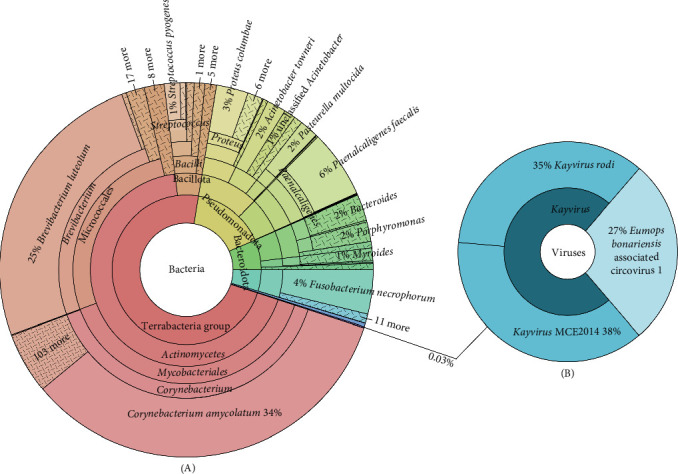
Metagenomic analyses of sample GB03 with bacterial classification on the left (A), and viral classification on the right (B). Host reads were removed prior to Kraken2 megatenomic classification. Plots were produced by KronaTools 2.8.1. Clades are coloured by major phylogenetic divisions based on the NCBI taxonomic database.

Metagenomic analyses revealed sequences with homology to the circovirus family (Figure 1B: *Eumops bonariensis* associated circovirus - Circovirus tzinaka; GenBank: OL704833). To reconstruct the full genome, non-host reads were assembled de novo using Trinity v2.13.2 [24]. Contigs were then compared against the nonredundant nucleotide database (NCBI) using BLAST [25] to identify putative circovirus contigs. To reconstruct the characteristic circular circovirus genome, we mapped sample reads back to the best matching Trinity contig and used the mapped reads for de novo circular assembly in Geneious Prime v2024.0.2 [26]. We annotated the genomes through open reading frame detection and alignment with known circovirus genomes (Supporting Information 2: Table S2).

Circovirus phylogenetic reconstruction is challenging due to their divergent and rapidly evolving genomes, particularly in the capsid (CAP) gene and intergenic regions [27]. To address this, we translated the more conserved replicase (REP) gene coding sequence and used amino acid alignments for phylogenetic analysis. For comparative material we used a database of 60 recognized circovirus species representing the phylogenetic breadth of the clade as compiled by the International Committee on Taxonomy of Viruses (ICTV; https://ictv.global/report/chapter/circoviridae/circoviridae/circovirus, accessed on 14^th^ of December 2023). We supplemented this with the top 21 BLAST hits (nine of which were ICTV duplicates) to include the most closely related potential isolates. After including the four generated circovirus sequences and an outgroup (*Cyclovirus adie*, GenBank Accession Number HQ738643), all 77 sequences were aligned using MAFFT [28]. A maximum likelihood credibility tree was constructed using Fasttree-2 [29] as implemented in Geneious Prime, applying the default Jones–Taylor–Thorton model.

### 2.4. Primer Development and Circovirus Detection by PCR

The nested circovirus primers of Halami et al. [30] did not reliably amplify all four known positive samples from the metagenomic library preparation, likely due to multiple primer mismatches despite their degenerate design. We therefore designed and tested four new primers targeting the same circovirus genomic region, specifically tailored to the novel virus (primer sequences in Supporting Information 3: Table S3). Using these primers, we performed nested PCR on DNA extracted from the six metagenomic samples' liver tissue. We used an initial touchdown protocol (10 cycles, 52 to 47°C) followed by 30 cycles at 47°C. Sanger sequencing (Eurofins Genomics, Edelsberg, Germany) confirmed the amplified products as the novel circovirus for all six samples.

Following this confirmation, we used regular PCR and agarose gel electrophoresis to screen for circovirus in liver samples from 188 hedgehogs that were previously investigated for a variety of putative pathogens (see [18] for details). This dataset includes hedgehogs from three different rescue centers in Belgium (Geraardsbergen, Merelbeke, and Oostende) and thus represents a large portion of the western part of Belgium. Samples with amplicons of the expected size (323 bps) were considered as positive for circovirus presence. Hedgehogs were classified as juvenile (less than 1 year) or adult using the jawline method [31].

### 2.5. Statistical Analyses of Association With HDD

Statistical analyses were performed in R version 4.2.3 (R Core Team, 2018). Exploratory data analysis revealed that both HDD and circovirus infection were predominantly found in adult hedgehogs, with most juveniles negative for both. This age-related bias could create a misleading association between HDD and circovirus in the full dataset. To address this, subsequent analyses focused exclusively on adult hedgehogs.

We used generalized linear mixed models (GLMMs) from the 'lme4′ package [32] to test for an association between HDD and circovirus occurrence. Models employed a binomial error distribution and as there could be biases between locations we included wildlife rescue center of origin as a random effect. Given the previously established association between *Borrelia burgdorferi* sensu lato and HDD [18], this pathogen was included as a fixed covariate in the GLMM analysis. Due to high collinearity, life stage was tested in a separate model.

## 3. Results

Illumina sequencing yielded 743 million reads across six samples (Supporting Information 1: Table S1). Following trimming and host read removal, metagenomic analyses classified the remaining reads as predominantly bacterial, with a minor viral component. Bacterial composition varied between individuals, though common commensal genera like *Staphylococcus*, *Streptococcus*, *Fusobacterium*, and others were detected in most samples (Supporting Information 4: Table S4 and Figure 1A). Aside from the previously identified *Corynebacterium* strains discussed in earlier studies on HDD [13, 18], no pathogenic bacteria relevant to HDD were detected. Viral classifications were less frequent, primarily representing phage-viruses, but also included a circovirus (Figure 1B and Supporting Information 4: Table S4). Considering the potential pathogenic role of circovirus on ulcer formation, and the fact that no circovirus has been described in hedgehogs to date, we investigated this novel finding in greater detail.

### 3.1. Circovirus Genome Assembly

**Figure 2:**
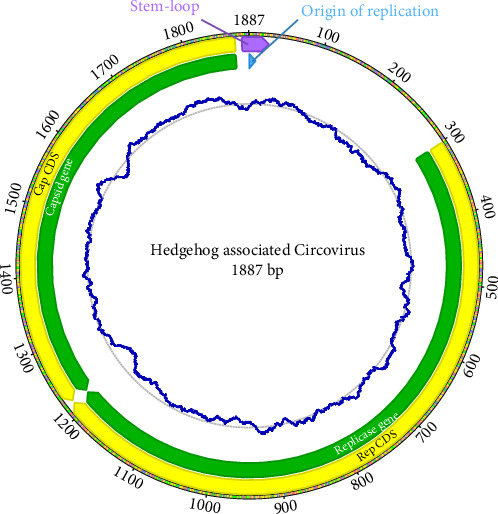
Full circular genome of the novel hedgehog associated circovirus generated in this study. The genome was 1887 bps long and included functional copies of the two circovirus genes; the CAP and the REP gene. Average GC content was 48.6% and it varied along the genome as indicated by the blue line. The stem-loop and origin of replication are indicated in purple and blue, respectively. The nonamer typical for circoviruses which functions as the origin of replication was CAGTATTAC. bps, base pairs; CAP, capsid; GC, guanine–cytosine; REP, replicase.

*De novo* assembly of the reference sample (GB03) yielded a complete circular genome assembly of 1887 base pairs (bps) with a guanine–cytosine (GC) content of 48.6% at an average coverage of 91X (Figure 2). Both REP and CAP genes were identified and translated into functional protein sequences. Three additional complete assemblies showed equal length and high sequence similarity (pairwise identities 98.1%–99.2%), and one individual (GB04) only included enough reads for reconstructing a partial genome of 1616 bps. All five sequences have been submitted to GenBank (PQ261155-PQ261159).

### 3.2. Phylogenetic Reconstruction

Phylogenetic reconstruction placed the novel circovirus within a well-supported clade containing circoviruses from diverse hosts (bats, carnivores, rodents, and a Tawny owl). It was most closely related to two circoviruses isolated from dogs in China [33]. The phylogeny highlights the lack of concordance between circovirus relationships and host species, emphasizing their ability to jump between highly divergent hosts.

### 3.3. Circovirus Prevalence Estimation and Statistical Analyses

All six metagenomic samples tested positive for circovirus via PCR amplification and Sanger sequencing. This included the two samples where cfDNA libraries lacked circovirus reads. Screening a larger dataset of 188 liver samples by agarose gel confirmation of the expected band size, revealed a high overall prevalence of 61% (110 positives), with a marked age-related difference (GLMM estimate = −3.5312, *z* = −7.050, *p*=1.79e − 12). Juvenile hedgehogs exhibited a significantly lower prevalence (27%, *n* = 90) compared to adults (92%, *n* = 98).

While an initial association between HDD and circovirus appeared strong across all samples (GLMM estimate = 2.83365, *z* = 3.619, *p*=0.000296), this was likely an artifact of the age bias in both disease (GLMM estimate = −2.0338, *z* = −3.527, *p*=0.000421) and infection (Supporting Information 5: Figure S1). When the analysis was restricted to a subset of 98 adult hedgehogs, the association between circovirus and HDD was no longer statistically significant (GLMM estimate = 1.26136, *z* = 1.090, *p*=0.276).

## 4. Discussion

**Figure 3:**
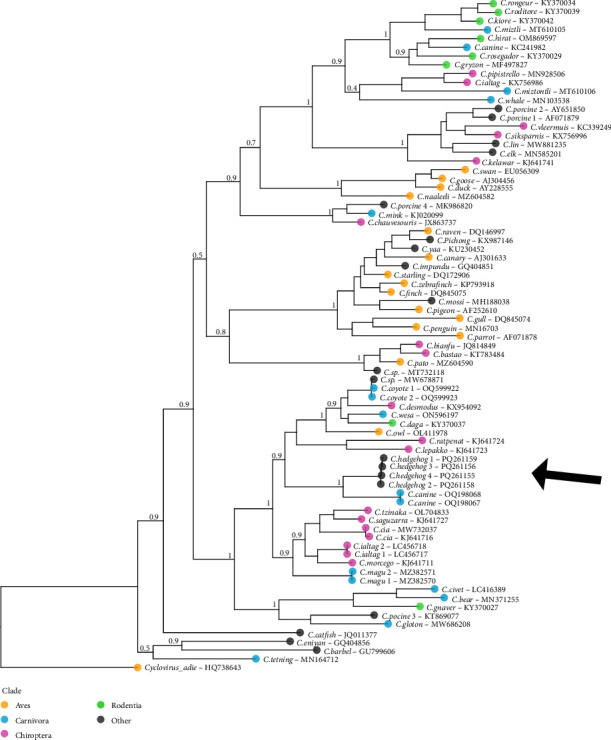
Phylogenetic reconstruction of the REP gene on an amino acid alignment of 77 samples spanning the phylogenetic width of the circovirus genus. The tree was rooted using *Cyclovirus adie*. Samples were coloured by host clade, focusing on the clades of aves, carnivora, chiroptera, and rodentia in which most circoviruses have been described. The four sequenced strains of the newly discovered *Circovirus hedgehog* are indicated by a black arrow. Internal node values of shallow clades were removed for figure clarity. REP, replicase.

Metagenomic analyses of blood samples from hedgehogs with HDD in Belgium revealed a novel circovirus species. Phylogenetically, it is most closely related to two circovirus strains isolated from dogs in China (Figure 3) and falls within a larger clade including porcine circovirus 3 (PCV3). This represents the first documented circovirus within the Eulipotyphla family that includes hedgehogs, shrews, and moles [34]. PCR amplification with species-specific primers confirmed a high prevalence of this circovirus in a biased sample set of sick or wounded hedgehogs brought to wildlife rescue centers.

The novel circovirus genome exhibits the characteristic structure found in most known circovirus species, including the circovirus-specific nonamer motif. Circoviruses are small, nonenveloped, single-stranded DNA viruses with circular genomes. The clade is known for their ability to infect a remarkably diverse range of hosts, yet most strains are species-specific. Most infections in both wild and domestic species are subclinical although they can cause a range of gastrointestinal, neurological, dermatological, and respiratory signs in their hosts, especially when found in conjunction with other pathogens (World Organisation for Animal Health (WOAH)—technical disease cards). Circoviruses can compromise host immunity and cause tissue inflammation, potentially increasing the likelihood of secondary infections, [35, 36] and thus circoviruses could plausibly impact HDD development in hedgehogs.

A very high prevalence of 92% of the novel circovirus was demonstrated in a cohort of hedgehogs of over 1 year of age that were brought to rescue centers (Supporting Information 5: Figure S1 and Supporting Information 6: Table S5). This is also the age class by far most affected by HDD in our dataset, however, no statistically significant associations were found between circovirus infections and HDD in this biased dataset of rescued hedgehogs. The lower prevalence of circovirus in the juveniles at 27% could indicate that the virus impacts juveniles differently [37]. Either hedgehogs get infected at a later age, or infections acquired at a young age lead to rapid mortality. Further studies, including random screening of wild hedgehog populations are needed to investigate any potential association between HDD, and circovirus prevalence in wild populations.

Circoviruses are characterized by rapid evolution and frequent host-switching events (Figure 3) [38]. This is also reflected in the large genetic distances between different circovirus species and strains. Rosario et al. [27] define species-level divergence at over 20% sequence difference, with most within-species diversity showing over 95% nucleotide sequence identity. The five strains of circovirus found in our dataset were all similar (above 98%), but highly divergent from the closest sequenced relative (circovirus canine) at only 66% sequence identity. The observation of two circovirus genomes within a single individual (GB03) suggests multiple transmission events rather than within-host evolution.

The high prevalence of the novel circovirus within Belgian hedgehog populations suggests several plausible epidemiological scenarios. As hedgehogs are not commonly tested for circovirus, it is possible that it has been endemic in hedgehog populations but remained undetected due to limited surveillance [39]. Alternatively, this circovirus could be a recent introduction to hedgehog populations through cross-species transmission [40, 41]. Understanding whether the virus is a long-standing endemic pathogen or an emerging infectious disease will require extensive historical and geographic sampling [42]. Further research into the virus's transmission dynamics, pathogenicity, and potential impact on hedgehog health are also crucial to better understand the current epidemiological situation.

cfDNA metagenomic sequencing detected a novel circovirus which would not have been detected by metabarcoding or circovirus specific PCR, highlighting the benefits of applying this technique for novel pathogen detection [4, 6, 43]. Although only a fraction of the total number of sequencing reads were used, the technique still detected circovirus in four out of six samples. However, false negatives are a common artefact of cfDNA studies [44], evident by the two additional cfDNA samples that did not contain circovirus reads but were subsequently found to be positive by nested PCR on liver samples from the same individual. This underscores that cfDNA offers great potential for novel pathogen detection, but results can be influenced by tissue choice, timing of sampling, and the possibility of false negatives.

## 5. Conclusion

This study revealed a novel circovirus species in Belgian hedgehogs. Phylogenetic analysis indicates a sister relationship to canine circovirus strains and the broader clade including porcine circovirus 3. While the virus exhibited high prevalence in a biased sample of sick and wounded hedgehogs, no significant association with HDD was found. Further investigation, including random screening of wild populations is warranted to elucidate links between HDD and circovirus infections.

## Data Availability

All data generated or analyzed during this study are included in this published article and its supporting information files. Illumina sequence data are available on the Sequence Read Archive under accession number PRJNA1091455 and reconstructed circovirus sequences are available under GenBank accession numbers PQ261155-PQ261159.

## Supporting Information

Supporting Information 1 Table S1: Sample table with a description of the cfDNA samples and the resulting reads per filtering step and assignment level.

Supporting Information 2 Table S2: Sample table of all the samples included in the Circovirus phylogenetic reconstruction.

Supporting Information 3 Table S3: List of primers developed and used in this study.

Supporting Information 4 Table S4: Results of the Kraken analyses with every sample represented by a different tab in the excel file.

Supporting Information 5 Figure S1: Pie-diagrams representing the correlation between Circovirus infection and HDD which is largely driven by the lower prevalence of both infections in juveniles. When restricting the analyses to adults the association was not significant.

Supporting Information 6 Table S5: Sample table of the rescue center hedgehog dataset with information on individual hedgehogs and their disease status.
