# Supplementary figures and images for: High Prevalence of a Novel Circovirus in the European Hedgehog (Erinaceus europaeus), a Common Species in Decline
Source: Transbound Emerg Dis. 2024 Nov 27;2024:4670252. doi: 10.1155/2024/4670252 (PMC12017114; doi:10.1155/2024/4670252)

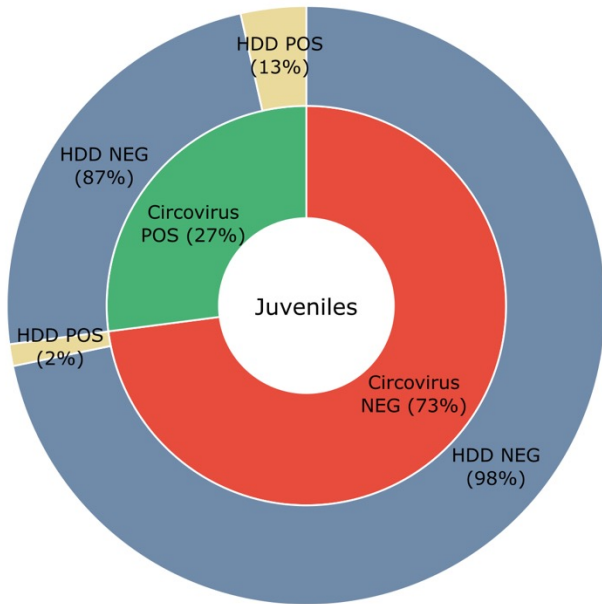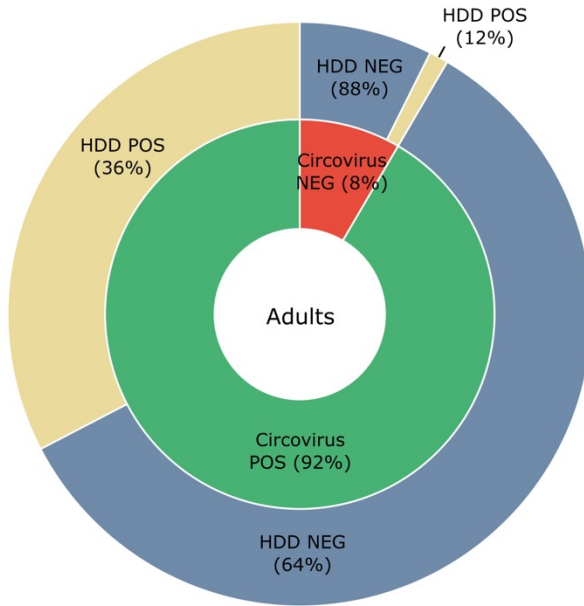

Supplement: Supporting Information 5 — Figure S1: Pie-diagrams representing the correlation between Circovirus infection and HDD which is largely driven by the lower prevalence of both infections in juveniles. When restricting the analyses to adults the association was not significant. [file 4670252.f5.pdf]
